# Study of Resveratrol’s Interaction with Planar Lipid Models: Insights into Its Location in Lipid Bilayers

**DOI:** 10.3390/membranes11020132

**Published:** 2021-02-14

**Authors:** Daniela Meleleo

**Affiliations:** Department of Biosciences, Biotechnologies and Biopharmaceutics, University of Bari “Aldo Moro”, via E. Orabona 4, 70126 Bari, Italy; danielaaddolorata.meleleo@uniba.it; Tel.: +39-080-5442775

**Keywords:** resveratrol, planar lipid membrane, cholesterol, channel-like event, capacitance

## Abstract

Resveratrol, a polyphenolic molecule found in edible fruits and vegetables, shows a wide range of beneficial effects on human health, including anti-microbial, anti-inflammatory, anti-cancer, and anti-aging properties. Due to its poor water solubility and high liposome-water partition coefficient, the biomembrane seems to be the main target of resveratrol, although the mode of interaction with membrane lipids and its location within the cell membrane are still unclear. In this study, using electrophysiological measurements, we study the interaction of resveratrol with planar lipid membranes (PLMs) of different composition. We found that resveratrol incorporates into palmitoyl-oleoyl-phosphatidylcholine (POPC) and POPC:Ch PLMs and forms conductive units unlike those found in dioleoyl-phosphatidylserine (DOPS):dioleoyl-phosphatidylethanolamine (DOPE) PLMs. The variation of the biophysical parameters of PLMs in the presence of resveratrol provides information on its location within a lipid double layer, thus contributing to an understanding of its mechanism of action.

## 1. Introduction

**Figure 1:**
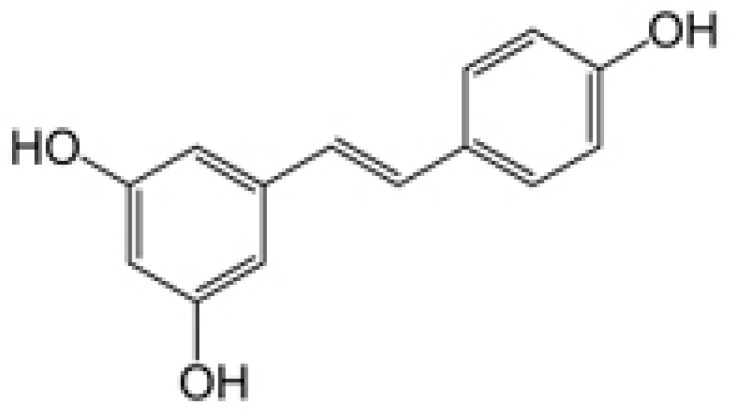
Structure of *Trans*-resveratrol.

Trans 3, 4′, 5-trihydroxystilbene, also known as *Trans*-resveratrol (Figure 1), is found in edible fruits and vegetables, particularly in grapes, blueberries, blackberries, and peanuts.

Resveratrol is also present in red wine, giving rise to the so-called “French paradox” by which, despite the fat-rich diets, mortality from coronary heart disease is lower in France than in other countries due to the inhabitants’ moderate consumption of red wine [1]. 

Besides its cardio-protective effects, resveratrol has shown a wide range of beneficial effects on human health, including anti-microbial [2], anti-inflammatory [3,4], anti-cancer [5,6], and anti-aging [7,8] properties. Some studies have shown that this polyphenol has a preventive effect on Alzheimer’s disease and dementia, due to its anti-inflammatory properties [9,10,11,12,13,14,15]. 

Numerous studies have been carried out in order to gain an understanding of the mechanisms underlying the beneficial effects of resveratrol [16]. The results of studies carried out by Yashiro et al. [17] and Voloshyna et al. [18] show that its cardio-protective effect may be due to resveratrol’s capacity to act as a modulator of lipoprotein metabolism. Indeed, resveratrol inhibits the oxidation of low-density lipoproteins and platelet aggregation, while increasing high-density lipoproteins [19]. It is known to be a scavenger of free radicals [20]. Several studies have shown that, when phospholipase A2 promotes the release of cytokines and NADPH oxidases that cause cell inflammation, resveratrol has proven to act as a good cell protective agent [21,22,23,24]. 

Although resveratrol’s beneficial effects on human health have been accepted, its molecular mechanism remains unclear. The biomembrane seems to be the main target of resveratrol, although its mode of interaction with membrane lipids and its location within the cell membrane are subject to debate. Resveratrol’s affinity for the lipid matrix is indicated by its poor water solubility (0.03 g/mL) and high partition coefficient (3.07 and 3.11 in 1,2-dipalmitoyl-*sn*-glycero-3-phosphocholine (DPPC) and 1,2-distearoyl-*sn*-glycero-3-phosphocholine (DSPC) liposomal membranes, respectively) [25]. 

Numerous studies have examined the interaction and location of resveratrol within the membrane utilizing different techniques and lipid models given that the cell membrane is a complex system. The results of these studies are not unique. Indeed, it has been either concluded that resveratrol is located in the hydrophilic headgroup region or, on the contrary, in the hydrophobic core.

The results of a study carried out by de Ghellinck and colleagues indicate that resveratrol accumulates between the lipid headgroups, causing conformational changes in the tilt angle of the lipid headgroups to a more upright orientation [26]. Incorporation and location of resveratrol in the hydrophilic headgroup region seem to depend on the lipid composition of the membrane model. The study carried out by Han and colleagues shows that, in 1,2-dioleoyl-sn-glycero-3-phosphatidylcholine (DOPC) liposomes, resveratrol could penetrate into the lipid membrane, locating mostly in the headgroup region rather than in the deeper region of the lipid double layer. In DOPC/sphingomyelin/cholesterol or sphingomyelin/cholesterol liposomes, resveratrol associates with the liposome surface without penetrating into the headgroup region. This different behavior shown by resveratrol may be due to a different packing of the lipid acyl carbon chains. In DOPC liposomes, the acyl carbon chains are less packed than in liposomes containing sphingomyelin and cholesterol [27]. Similar results have also been obtained by Cardia and colleagues, who studied the interaction of resveratrol with soybean phosphatidylcholine (P90G) liposomes by ^1^H NMR spectroscopy. Resveratrol is located in the region of the phosphocholine headgroup. Quantitative data on the incorporation of resveratrol at both the liposome preparation stage and by preformed liposomes suggest that the amount of resveratrol incorporated into P90G liposomes was about 20 mM, which corresponds to a 150-fold increase with respect to the solubility of resveratrol in water. These experimental data show that resveratrol diffuses through lipid bilayers [28]. Using liposomes made from saturated phosphatidylcholine (DPPC) and cholesterol (CHOL) or positively charged derivates of cholesterol (DC-CHOL), Bonechi et al. found that resveratrol associates with the surface of liposome containing CHOL, as found by Han et al. [27], while the polyphenolic compound inserts more deeply into cationic liposomes [29].

On the other hand, some authors state that resveratrol is located in the hydrophobic core. The results of a study carried out by Balanč and colleagues indicate that resveratrol is incorporated in the inner part of the liposome membrane [30]. In agreement with the results obtained by Balanč are those presented by Fabris and colleagues who, using phosphatidylcholine liposomes of various chain lengths, show that the hydroxyl groups of resveratrol are located in the lipid region of the bilayers close to the double bonds of polyunsaturated fatty acids and that the longer the acyl chains, the more stable and less perturbable the bilayers [25]. Similar results have also been obtained by Brittes and colleagues [1]. In addition, Neves and colleagues show that resveratrol induces phase separation and formation of liquid-ordered domains in liposomes made of egg L-α phosphatidylcholine, cholesterol and sphingomyelin. Besides, this polyphenol stabilizes the membrane bilayers of the liposome locating in a deeper region of the membrane and adopting a vertical position in the nonpolar region of the membrane, with two hydroxyl groups in the interfaces [31,32]. 

The penetration of a drug into the lipid bilayer is closely related to its antioxidant activity [33,34] because it affects membrane fluidity and polarity. Indeed, numerous studies indicate that increases or decreases in membrane fluidity are responsible for the antioxidant effects of many drugs. Antioxidant drug molecules can trap free radicals or hamper their diffusion by fluidifying [1,25,35] or stiffening [36,37,38] the membrane, respectively. Resveratrol is able to fluidize and permeate the membrane, suggesting that it is a good antioxidant agent. Besides, the ability of resveratrol to penetrate into the membrane may depend on differently packed lipid, fluid or gel phases [1]. This behavior of resveratrol could explain the controversies regarding its location within the membrane. 

In this paper, we report a systematic investigation of resveratrol incorporation into planar lipid membranes (PLMs) and formation of channel-like events using electrophysiological measurements, a new technical approach for the study of resveratrol’s membrane activity, aiming to contribute to understanding the action of resveratrol on membranes, by varying PLM electrical parameters (capacitance and conductance) when the polyphenolic compound interacts with it. PLMs of different composition were used for the following reasons: they are a convenient tool to use for protein or drug incorporation, as they are a less complex membrane model system than plasma membranes; they complement studies performed with liposomes.

Incorporation into membranes and channel-like event formations are a test of resveratrol’s affinity for the lipid core of PLMs, and, albeit indirectly, they can provide information on its location within the lipid double layer.

## 2. Materials and Methods

### 2.1. Reagents and Equipment 

**Figure 2:**
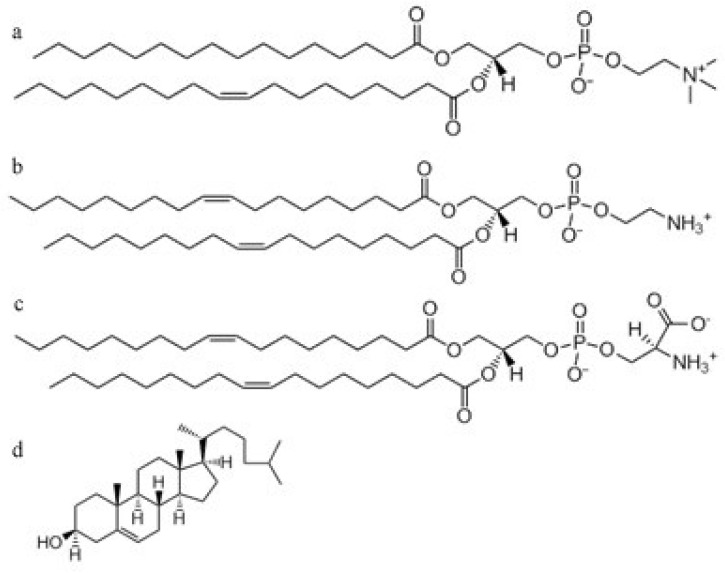
Molecular structures of (**a**) palmitoyl-oleoyl-phosphatidylcholine (POPC), (**b**) dioleoyl-phosphatidylethanolamine (DOPE), (**c**) dioleoyl-phosphatidylserine (DOPS), (**d**) and Cholesterol (Ch).

Salts and other basic chemicals were bought from Sigma (Munich, FRG, analytical grade). The phospholipids were purchased as follows: palmitoyl-oleoyl-phosphatidylcholine (POPC) from Avanti Polar Lipids, (Alabaster, AL, USA); dioleoyl-phosphatidylserine (DOPS); dioleoyl-phosphatidylethanolamine (DOPE); Cholesterol (Ch) from Sigma (Munich, FRG), resveratrol was from Farmalabor^®^ S.r.l. (Canosa of Puglia, Italy). Figure 2 reports the molecular structures of the lipids used in this study. The volumes (Vl) of phospholipids are similar (Vl = 1231.2/1228/1254 Å^3^ POPC/DOPE/DOPS, respectively) [39,40,41], while their shapes are different. Indeed, POPC and DOPS are classified as cylindrical lipids, and DOPE as a conical lipid [42].

**Figure 3:**
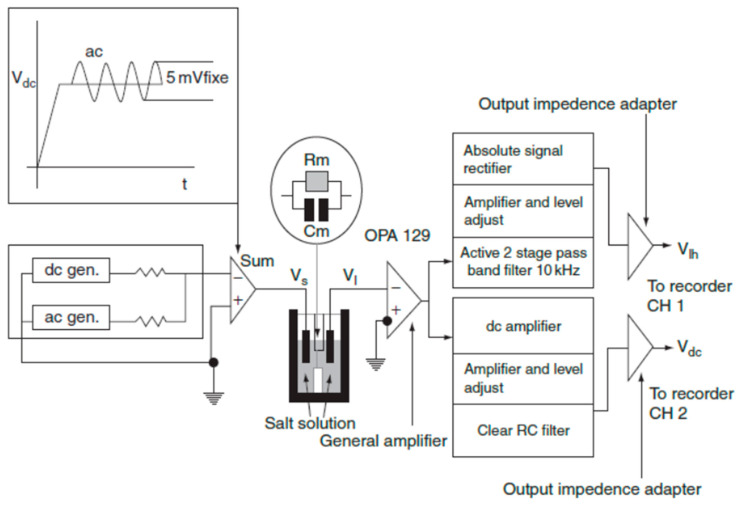
Experimental set-up during investigations into resveratrol channel-like incorporation.

Single-channel measurements: the membrane current was monitored with an oscilloscope and recorded on a chart recorder for further analysis by hand. A Teflon chamber made up of two aqueous compartments connected by a small circular hole with a diameter of 300 µm was used. The *cis* and *trans* chambers were connected to the amplifier head stage by Ag/AgCl electrodes in series with a voltage source and a highly sensitive current amplifier (OPA 129). The single-channel instrumentation had a time resolution of 1–10 ms depending on the magnitude of the single-channel conductance. Figure 3 reports the experimental set-up used to study the interaction of resveratrol with PLMs. The polarity of the voltage was defined according to the side where resveratrol was added (the *cis* side). A trans-negative potential (indicated by a minus sign) means that a negative potential was applied to the *trans* side, the compartment opposite the one where resveratrol was added. These experiments are considered to be a sensitive tool for the study of channel-forming substances [43,44].

### 2.2. Experimental

#### 2.2.1. Preparation of Resveratrol Solution

A stock solution of *Trans*-resveratrol (Res, M.W. 228.25) was prepared by dissolving resveratrol powder (16 mg) in 1000 µL of dimethyl sulfoxide (DMSO), stirring for 3 min to obtain a concentration of 70 mM. From this solution, 143 µL were withdrawn and diluted in 857 µL of bidistilled sterile water under stirring for 3 min to obtain a concentration of 10 mM. Both solutions were stored in dark glass vials at −20 °C until use, and 4 or 8 µL of the second solution was added to the *cis*-side of the membrane, to obtain the final concentration of 10 or 20 μM, respectively. 

#### 2.2.2. Preparation of PLMs

Channel-like activities were recorded in PLMs of POPC or POPC:Ch (65:35, *w*/*w*) or DOPS:DOPE (1:2, *w*/*w*) in 1% of n-decane. Bilayers were formed across a 300-μm hole in a Teflon partition separating two Teflon chambers (volume 4000 µL) which held symmetrical 0.1 M KCl solutions, pH = 7, temperature 23 ± 1 °C. The aqueous solutions were used unbuffered. The salts used in the experiments were of analytical grade. The Müller-Rudin or painted technique was used to form PLMs with lipids solubilized in n-decane [45,46]. Briefly, a small volume of 0.5–1 µL of lipid solution is applied through a micropipette directly onto the hole of the Teflon set; a PLM forms in ~10 min after draining the excess solvent into the aqueous bathing solution. In all experiments performed, the conductance and capacitance of each membrane was tested by applying a voltage of ±120 mV for 15–20 min under stirring to ensure that the membrane was stable.

#### 2.2.3. Determination of Conductance of Channel-Like Events

To determine conductance, we measured the amplitude of channel-like events by hand. The single channel data, filtered at 300 Hz, were obtained from at least three experiments (more than 100 single channel-like events) performed on different days. A histogram of the conductance amplitude distribution for each experiment was constructed and fitted by a Gaussian distribution function. Results are expressed as central conductance ±standard error (Λ_c_ ± SE) and were evaluated by analysis of variance (ANOVA-Tukey test) and Student t test. A value of *p* < 0.05 was considered significant. The Gaussian distribution function, ANOVA test, Student t test, and the fitting procedures were performed using the GraphPad Prism 3 software (GraphPad Prism^TM^ version 3.0, GraphPad Software, San Diego, CA, USA). 

To determine the frequency (number of channel-like events in 60 s), any detection of channel events was counted as successful. Results are expressed as frequency ± standard deviation (F ± SD).

#### 2.2.4. Determination of PLM Capacitance

The method used to determine PLM capacitance was as reported in a previous study [47]. Briefly, membrane capacitance was calculated using a calibration curve obtained by simulating the membrane capacitance with a discrete set of capacitances of known values, *C_n_*, and measuring the corresponding output voltage, *V_lh_*. The data obtained were fitted by the formula:

$$V_{lh}=\frac{A\times C_{n}}{\left(B+C_{n}\right)},$$

in which *A* and *B* are free parameters to be estimated by the fitting procedures. The values of parameters *A* and *B* were used to transform the *V_lh_* value into capacitance data.

## 3. Results

### 3.1. Membrane Stability

Many studies have shown that resveratrol interacts with membrane lipids due to its lipophilicity. Resveratrol’s localization in membranes or membrane models is a matter of debate.

In this study, we used a classic electrophysiological method to investigate resveratrol’s microscopic incorporation at concentrations of 10 and 20 μM, in PLMs of different composition. Channel-like event formation indicates that molecules of a substance are incorporating into the membrane. PLMs of different composition were used to see whether resveratrol is able to incorporate into PLMs and to form conductive units, and under what experimental conditions. With this aim, POPC was used as a zwitterionic membrane, POPC:Ch as a neutral mixed membrane and DOPS:DOPE as a mixed membrane with diluted charges.

First of all, we tested PLM stability by applying a voltage of ±120 mV for 10–15 min under stirring and by monitoring the constant values for PLM conductance and capacitance. Preliminarily, to exclude any non-specific and destabilizing effects of DMSO per se on the PLMs used, we performed experiments by adding 4 or 8 μL of a H_2_O:DMSO (6:1) mixture to the medium, on the *cis* side of the membrane. In many different experiments and independently of lipid composition, the PLMs were stable even when applying a voltage of ±100 mV for 12 h under stirring and monitoring the constant values for PLM conductance and capacitance.

### 3.2. Resveratrol Interaction with POPC PLMs

**Figure 4:**
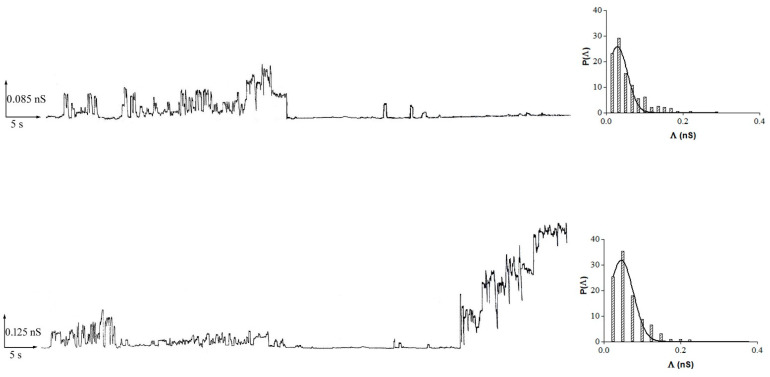
Resveratrol channel-like activity in POPC planar lipid membranes (PLMs). Representative traces illustrating channel activity of resveratrol in membranes made up of POPC with associated histograms of the conductance fluctuations. The histograms of the probability, P(Λ), for the frequency of a given conductivity unit were fitted by a Gaussian which is shown as a solid curve. Experiments were performed in the presence of 10 μM (top trace) and 20 μM (bottom trace) of resveratrol added to the *cis* side, while the aqueous phase contained 0.1 M KCl (pH 7) and T = 23 ± 1 °C. Applied voltage was set to 60 mV (top trace) and 40 mV (bottom trace).

In many different experiments on POPC PLMs, the addition of 10 or 20 μM of resveratrol to the *cis* side of the medium facing the membrane was performed at the applied voltage of 100 mV (addition voltage). After different lag times depending on the resveratrol concentration, channel-like activity appears as non-random discrete events that fluctuate between conductive or non-conductive states compatible with channel-type openings and closures with different conductance levels and frequencies. After the first channel-like event, alternating periods of channel-like activity can be observed, during which the number of events can be rigorously analyzed, followed by quiescent periods and periods of paroxystic channel-like activity that often lead to membrane destabilization until rupture. Figure 4 shows a typical example of channel-like event recordings with associated histograms of the conductance fluctuations.

In experiments at the 10 μM resveratrol concentration, channel-like activity appears after about 60 minutes’ lag time at an applied voltage of 100 mV. After the first channel-like event, the applied voltage can be lowered as far as 60 mV and channel amplitude can be monitored. The channel-like activity was registered at applied voltages of ±60, ±80, ±100, and ±120 mV, each applied for 1 h, starting from 60 mV. 

By doubling the resveratrol concentration to 20 μM, the lag time reduced to 20 min, after which channel-like activity appeared at an applied voltage of 100 mV, manifesting in a paroxystic manner. Paroxystic activity occurred even when the applied voltage was lowered to 80 mV; therefore, the applied voltage was lowered to 60 mV and channel-like activity appeared as discrete current fluctuations with different conductance levels. Channel-like activity was monitored at applied voltages of ±40 and ±60 mV, each applied for 1 h, starting from 60 mV. 

Table 1 summarizes the central conductance values (Λ_c_ ± SE), obtained by fitting the experimental data with a Gaussian function (Figure 4), and frequency values (F ± SD) at the two different concentrations of resveratrol. Interestingly, at the 10 µM resveratrol concentration, the frequency values were higher at negative applied voltages than at positive ones, indicating a higher turnover of channel-like events.

Another parameter that we calculated is the duration of observed channel-like events. The duration of channel-like events was within a range from 1.25 to 2.25 s and from 0.75 to 1.25 s when the resveratrol concentration was 10 and 20 μM, respectively. The lifetime for two resveratrol concentrations, although not significantly different, indicates that channel-like events were more stable at lower resveratrol concentrations than at higher ones. 

The results obtained with POPC PLMs indicate that resveratrol is able to penetrate into the hydrophobic core of PLMs and to form channel-like events. The lag time, the applied voltages at which the channel-like activity manifests, and the shorter lifetimes decrease on increasing resveratrol concentration, in agreement with its poor water solubility and its partition coefficient and with the concept that an appropriate resveratrol/lipid ratio, is required to permeabilize the membrane. 

Our experimental apparatus can measure conductance and capacitance simultaneously. Capacitance is related to the property of a membrane to act as a capacitor and is inversely proportional to its thickness. The capacitance parameter is considered to be the best tool for probing the stability and formal goodness of the double lipid layer before and after any experiment concerning the incorporation of a different substance can be conducted [47]. 

In all experiments, before the addition of resveratrol, the basic capacitance of the POPC PLMs was in the range of 0.27–0.28 µF/cm^2^, remaining constant. After the addition of resveratrol, we observed that the capacitance decreased during the lag time, then increased when the first channel-like events appeared, reaching higher values than those of basic capacitance; these values remain almost constant all the way through to the end of the experiment. This behavior was independent of the resveratrol concentrations used in this study. 

Table 2 reports the mean capacitance (C ± SE) values calculated at different times before and after the addition of resveratrol. To standardize the results, capacitance was measured just before the addition of resveratrol (T0), 70% of the way through the lag time (T1), at the end of the lag time when the channel-like activity appears (T2), and after an average of 1 h from the first channel-like event when the capacitance signal was almost constant (T3). The capacitance variation observed could be due, initially, to resveratrol adsorption to the planar lipid bilayer and, subsequently, to its insertion into the bilayer once an appropriate resveratrol/lipid ratio had been reached.

### 3.3. Resveratrol Interaction with POPC:Ch PLM

The addition of cholesterol to the POPC solution used to form membranes results in channel-like activity, indicating that the sterol does not prevent the insertion of resveratrol molecules into PLMs made up of POPC:Ch. 

The addition voltage of resveratrol was 100 mV both for experiments at a concentration of 10 μM and for those at a concentration of 20 μM. The lag time and applied voltage in which the channel-like activity appears depend on the resveratrol concentration used in the two series of experiments. 

**Figure 5:**
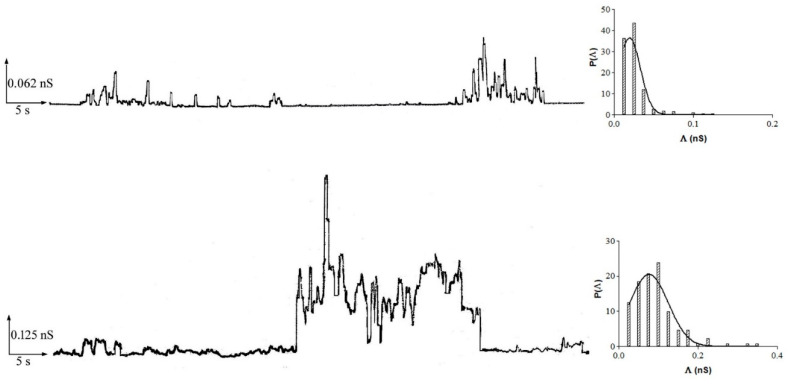
Resveratrol channel-like activity in POPC:Ch PLMs. Representative traces illustrating channel activity of resveratrol in membranes made up of POPC:Ch (65:35, w:w) with associated histograms of the conductance fluctuations. The histograms of the probability, P(Λ), for the frequency of a given conductivity unit were fitted by a Gaussian which is shown as a solid curve. Experiments were performed in the presence of 10 μM (top trace) and 20 μM (bottom trace) of resveratrol added to the *cis* side, while the aqueous phase contained 0.1 M KCl (pH 7) and T = 23 ± 1 °C. Applied voltage was set to 80 mV (top trace) and 40 mV (bottom trace).

After the addition of resveratrol to the *cis* side of the medium facing the membrane, channel-like activity manifests, first of all, as a paroxystic activity then characterized by current jumps compatible with channel-type openings and closures with different conductance levels followed, sometimes, by quiescent periods. Figure 5 shows an example of chart recordings of resveratrol’s channel-like activity when incorporated into POPC:Ch PLMs at the applied voltage of 80 and 40 mV with associated histograms of the conductance fluctuations.

In the set of experiments at 10 μM of resveratrol, channel-like activity appears after a lag time of 6–7 h at an applied voltage of 120 mV (activation voltage) as a paroxystic activity, after which the applied voltage was lowered down as far as 80 mV where the channel-like event amplitude could be monitored. The channel-like activity was recorded at applied voltages of ±80, ±100, and ±120 mV, each applied for 1 h, starting from 80 mV.

In the set of experiments at 20 μM of resveratrol, channel-like activity manifests as a paroxystic activity after a lag time of 2 h at an applied voltage of 100 mV followed by membrane rupture. After PLM breakage and withdrawal, the channel-like activity occurred spontaneously after a lag time of about 15 min at an applied voltage of 40 mV. The channel-like activity was monitored at applied voltages of ±40 mV, each applied for 90 min, starting from 40 mV. The application of voltages higher than ±40 mV (±60 and ±80 mV) determined paroxystic activity followed by destabilization of the membrane.

Table 3 reports the biophysical and statistic parameters (Λ_c_ ± SE and F ± SD, respectively) of the channel-like events obtained under the two different experimental conditions. It is important to note that, at a resveratrol concentration of 10 μM, the central conductance and frequency values seem to be independent of applied voltages both at positive and at negative voltages.

The comparatively higher lag times and applied voltages observed in these experimental sets than those for POPC PLMs may be due to the effect of cholesterol on acyl carbon chains that are more closely packed. Resveratrol penetrates the hydrophobic core of the membrane less easily.

As was done for POPC PLMs, we calculated the lifetime of channel-like events formed by resveratrol. The duration of channel-like events was within a range from 1.25 to 1.75 s when the resveratrol concentration was 10 μM, while, at 20 μM, the duration of channel-like events was 1.75 s at applied voltages of ±40 mV. 

The capacitance behavior recorded in the experiments with POPC:Ch PLMs was similar to that observed for POPC PLMs at the two different resveratrol concentrations. Table 4 reports the mean capacitance values at four different times, T0, T1, T2, and T3. It is interesting to note that the low values of capacitance measured at 70% of lag time (T1) could be due to resveratrol adsorption to the POPC:Ch PLM before its insertion into the bilayer, similarly to that observed for POPC PLMs.

### 3.4. Resveratrol Interaction with DOPS:DOPE PLMs

In order to test the role of the polar head and the effect of negative membrane charge on resveratrol incorporation and channel-like events, we carried out experiments using PLMs made up of DOPS:DOPE (1:2, *w*/*w*) in which the negative charge is diluted. This membrane is characterized by the presence of the net negative charge of DOPS and the neutral DOPE, a non-lamellar-forming lipid.

The resveratrol concentrations tested were 10 and 20 μM. The addition of resveratrol to the *cis* side of the medium facing the membrane was made at an applied voltage of 100 mV, using the same protocol as that described for POPC and POPC:Ch PLMs.

In all experiments with DOPS:DOPE membranes and independently of the resveratrol concentration, no current fluctuations were observed for many hours using a wide range of applied voltages. No channel-like activity occurred after PLM breakage and the formation of the second membrane by painting the lipid solution present around the hole.

In addition, in these experimental sets, we monitored capacitance. In all experiments, before the addition of resveratrol, the basic capacitance of the PLMs remained constant in the range of 0.26–0.28 µF/cm^2^. After the addition of resveratrol, we observed that the capacitance decreased during the lag time until reaching a minimum value that remained constant until the end of the experiment. This behavior was observed both at 10 and 20 μM of resveratrol. As in the experiments with POPC and POPC:Ch PLMs, we calculated the mean capacitance values at the different times, T0, T1, T2, and T3, which are reported in Table 5. The capacitance values remain low (0.11 ± 0.03 µF/cm^2^) even after PLM breakage and formation of the second membrane. 

These results indicate that resveratrol is unable to incorporate into PLMs made up of DOPS:DOPE and to form channel-like events, probably due to the presence of DOPE, a non-lamellar-forming lipid, as resveratrol may induce a negative curvature of the membrane. The combination of these factors prevent resveratrol incorporation into PLMs; however, we cannot exclude an effect of negatively charged lipid DOPS. 

## 4. Discussion

In recent decades, natural derivatives have been the subject of intensive studies regarding their biological and pharmacological properties. Resveratrol is known for its numerous beneficial effects on human health, even though its mechanisms of action are still unclear. Due to its lipophilicity and poor water solubility, the lipid membrane is the principal target of resveratrol. 

An important aspect of resveratrol’s interaction with the membrane is that it induces changes in the membrane’s biophysical properties. The resveratrol molecule may induce changes either to the interfacial structure or to the chain region, thus modifying membrane packing. Therefore, it is very important to be able to clarify the exact location in or on the membrane. 

In this work, by simultaneously measuring membrane conductance and capacitance, we show that resveratrol interacts with a planar lipid membrane and the mode of interaction is due to the bilayer’s lipid composition. To our knowledge, this is the first study in which resveratrol’s membrane activity has been monitored by electrophysiological measurements, which are not so frequently used for the characterization of drug-membrane interactions and by PLMs that complement studies performed with liposomes.

As is well known, conductance is related to the ionic current through the bilayer when a substance (a protein, peptide, or drug) incorporates into the membrane forming conductive units that span the bilayer, while capacitance is related to the property of a membrane to act as a capacitor; indeed, the lipid bilayer is an insulator separating two electrolytic media. Capacitance is directly proportional to the area of the membrane and inversely proportional to its thickness. Capacitance also depends on the characteristics of the lipids used as the insulating material. 

Several studies show that the structure of lipids determines the biophysical properties of membrane (e.g., organization in bilayers, fluidity, hexagonal phases, etc.) [48]. In our work, we used POPC that is prone to form a bilayer [49,50], cholesterol that has effects on membrane fluidity, and DOPE that is prone to form inverse hexagonal phases [49,50]. During the interaction of resveratrol with the planar lipid bilayer, the variation in capacitance can help to shed light on its mechanism of action. 

Our results show that resveratrol incorporates into PLMs made up of POPC and forms transient channel-like events in which lag time depends on its concentration. The lag time is the time at which a conductance variation first occurs after resveratrol addition. The different duration of lag time may be due to reaching an appropriate resveratrol/lipid ratio. Once a threshold resveratrol/lipid value has been reached, resveratrol forms transient conductive units permeabilizing the membrane. The capacitance variation, induced by adding resveratrol, may be due to: adsorption of resveratrol (capacitance decrease) occurring before formation of conductive units and/or formation of channel-like events (capacitance increase) across the membrane. The paroxystic activity reported above may be due to the collapse of the conductive units inducing rapid flip-flopping of the membrane lipids. It is important to note that both the lag time and the applied voltages at which the paroxystic activity appears depend on the resveratrol concentration, thus strengthening the concept that an appropriate resveratrol/lipid ratio has been reached. 

The addition of cholesterol, a known component of the cellular membrane, to PLMs made up of POPC increases the lag time compared to that observed for experiments with POPC PLMs, regardless of the resveratrol concentration used. The capacitance behaves in a similar fashion to that observed for experiments with PLMs in the absence of cholesterol, decreasing immediately after resveratrol addition and increasing when the channel-like activity appears. Therefore, also in POPC:Ch PLMs, the formation of channel-like events comes after the resveratrol adsorption phase. However, the higher lag times than those obtained for POPC PLMs would seem to indicate that cholesterol makes it more difficult for resveratrol to incorporate into PLMs and to form conductive units, probably due to the tighter packing of the phospholipid tails. 

According to some studies, the β-hydroxyl group attached to C3 of the cholesterol molecule, is located in the headgroup region of POPC, while the isooctyl chain, attached to C17, is located deep within the hydrophobic core of the bilayer close to the double bonds of fatty acids [24]. Some authors [1,25] have shown that resveratrol adopts the same position in the membrane as cholesterol. Besides, the higher applied voltage (120 mV) needed to obtain channel-like activity, compared with POPC PLMs, seems to confirm this idea. 

Our results appear to be in line with those obtained by other authors [25,29,51] who have shown that resveratrol penetrates into the hydrophobic core of the bilayer close to the double bonds of polyunsaturated fatty acids. 

This mode of interaction with lipids might explain the anti-peroxidation effects displayed by resveratrol, because it protects the phospholipids from oxidation reactions, acting as an electron donor to a free radical, neutralizing it and inhibiting its capacity to damage the cell membrane [52]. 

In contrast with its effects in neutral PLMs, resveratrol does not interact with DOPE PLMs to which negatively charged lipid DOPS have been added. To our knowledge, the interaction of resveratrol with the lipid system containing these phospholipids has never previously been studied.

In the experiments with DOPS:DOPE PLMs, the capacitance decreases after resveratrol addition reaching a minimal value that is quite constant until the end of the experiment. 

The behavior of the capacitance and the absence of channel-like activity indicate that resveratrol remains on the membrane surface and is unable to incorporate into the PLM. This behavior may be due to the chemical characteristics of the lipids forming the bilayer and/or to the effect of resveratrol on the membrane surface, which promote the formation of the inverse hexagonal phase and of membrane negative curvature. 

Several studies have shown that the nature and molecular shape of lipids determine their intrinsic tendency to form distinct phases [49] and to affect membrane curvature. Phosphatidylethanolamine (PE) has a truncated cone shape because its headgroup has a smaller area than the cross-section of its hydrocarbon chains, while phosphatidylcholine (PC) has a cylindrical molecular shape. Because of these features, PE is prone to form an inverse hexagonal phase and PC bilayer. Besides, dioleoyl lipids with phosphatidylethanolamine headgroups adopt a negatively-curved surface because the PE headgroup is smaller than the PC headgroup [53], and they are able to interact most with the glycerol and phosphate groups of neighboring lipids, probably due to the differing hydration properties influencing the curvature [54].

On the other hand, it has been demonstrated that many substances that are active at the membrane interface induce membrane curvature, namely local or global deformations of the membrane. 

Numerous studies show that there are many substances, interfacially active peptides [55,56,57] and non-peptide compounds that can influence membrane curvature, and vice versa. Yesylevskyy and colleagues have shown that the permeability of the model lipid membrane for cisplatin and gemcitabine depends on the curvature [58]. Barry and colleagues showed that curcumin, a lipophilic drug, binds to dimyristoyl-phosphocholine (DMPC) with a transbilayer orientation, anchoring to the bilayer by a hydrogen bond to the phosphate group, while, in dipalmitoleoyl-glycero-phosphoethanolamine (DiPOPE) vesicles, it promotes and stabilizes the formation of the inverted hexagonal (H_II_) phase. According to the authors, this feature of curcumin is indirect evidence that it induces negative membrane curvature [59]. 

On the other hand, primaquine, a potent antimalarial agent, increases the phase transition temperature (T_H_) of Lα and reduces the transition enthalpy of palmitoyl-oleoyl-phosphatidylethanolamine (POPE) vesicles, indicating that primaquine promotes positive membrane curvature and stabilizes the fluid phase of POPE vesicles [60]. 

All these studies indicate that many substances, peptides, and drugs induce modifications of a membrane’s biophysical properties caused by the chemical characteristics of the substances in question. In line with this concept, our work shows that resveratrol interacts with model membranes, modifying their biophysical parameters for different lipid compositions. Studies on drug-membrane interaction are very important in order to understand and clarify their mechanism of action.

## 5. Conclusions

**Figure 6:**
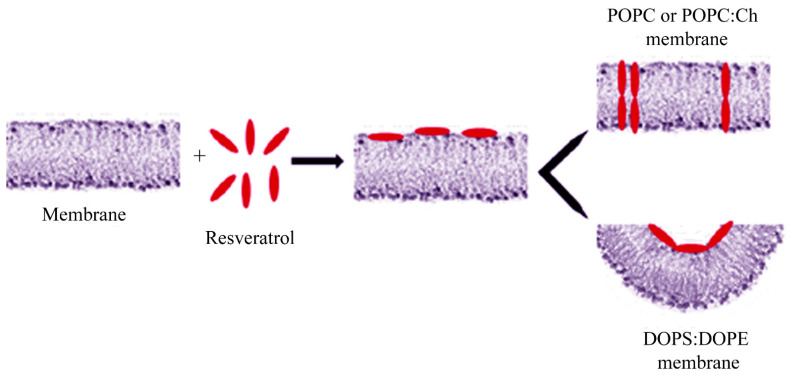
Schematic model of the resveratrol interaction with PLM at different lipid composition. Resveratrol adsorbs onto the membrane surface, regardless to the PLM composition. In POPC or POPC:Ch PLMs (top), resveratrol inserts and assembles into membrane forming conductive units, once an appropriate resveratrol/lipid ratio has been reached. In DOPE:DOPS PLMs (bottom), resveratrol inducing negative curvature of membrane is unable to insert into it.

Our results indicate that resveratrol interacts with zwitterionic and neutral PLMs, by incorporating into PLMs and forming transient conductive units, whereas it is unable to interact with DOPE:DOPS PLMs. In light of this, we hypothesize a model in which its fundamental steps are: (1) adsorption of resveratrol onto the membrane surface. This step is common to the neutral PLMs and to the negatively-charged PLMs used in this study; and (2) in neutral PLMs, formation of transient conductive units made up of molecules of resveratrol oriented with their long axis along the membrane normal and assembled together (Figure 6). The incorporation and channel-like events formation indicate that resveratrol is located in the hydrophobic core of the bilayer. These results, obtained by simultaneously monitoring membrane conductance and capacitance, may help to clarify the mechanism of action by which resveratrol performs its beneficial effects on human health, such as its antioxidant or anti-microbial properties and its scavenging of lipid radicals.

## Figures and Tables

**Table 1:** Characteristic parameters of resveratrol channel-like event in POPC PLM. The mean conductance (Λc ± SE) and frequency (F ± SD) of resveratrol channel-like events at different applied voltages. The minimum and maximum number of channel-like events considered (N) out of a total number of channel-like events considered (Nt) was: 130 < N < 375, Nt = 1640, at the resveratrol concentration of 10 µM. The minimum and maximum number of channel-like events considered (N) out of a total number of channel-like events considered (Nt) was: 280 < N < 622, Nt = 1757, at the resveratrol concentration of 20 µM.

	[Res] = 10 µM		[Res] = 20 µM	
Vs mV	Λc ± SE nS	F ± SD	Λc ± SE nS	F ± SD
120	0.012 ± 0.003	12.28 ± 1.08		
100	0.022 ± 0.002	13.90 ± 1.48		
80	0.019 ± 0.006	14.47 ± 0.74		
60	0.029 ± 0.004	9.88 ± 0.52	0.034 ± 0.001	12.20 ± 0.55
40			0.046 ± 0.003	13.03 ± 0.52
−40			0.054 ± 0.003	12.50 ± 1.01
−60	0.030 ± 0.003	16.97 ± 0.98	0.034 ± 0.001	9.71 ± 0.51
−80	0.016 ± 0.008	30.28 ± 4.16		
−100	0.020 ± 0.001	21.93 ± 1.35		
−120	0.016 ± 0.0006	18.78 ± 2.21		

**Table 2:** Capacitance variation in POPC PLM. Mean values of the membrane capacitance (C ± SE) calculated at T0 (just before the addition of resveratrol), T1 (at the 70% of lag time), T2 (at the end of lag time), and T3 (after an average of 1 h from first channel-like event appearance). The mean value was obtained from at least three experiments.

Time	C ± SE µF/cm^2^ [Res] = 10 µM	C ± SE µF/cm^2^ [Res] = 20 µM
T0	0.27 ± 0.01	0.28 ± 0.01
T1	0.11 ± 0.002	0.10 ± 0.003
T2	0.28 ± 0.02	0.29 ± 0.01
T3	0.30 ± 0.02	0.31 ± 0.02

**Table 3:** Characteristic parameters of resveratrol channel-like event in POPC:Ch PLM. The mean conductance (Λc ± SE) and frequency (F ± SD) of resveratrol channel-like events at different applied voltages. The minimum and maximum number of channel-like events considered (N) out of a total number of channel-like events considered (Nt) was: 186 < N < 516, at the resveratrol concentration of 10 µM. The minimum and maximum number of channel-like events considered (N) out of a total number of channel-like events considered (Nt) was: 265 < N < 337, Nt = 602, at the resveratrol concentration of 20 µM.

	[Res] = 10 µM		[Res] = 20 µM	
Vs mV	Λc ± SE nS	F ± SD	Λc ± SE nS	F ± SD
120	0.020 ± 0.001	11.10 ± 0.49		
100	0.020 ± 0.001	9.63 ± 0.62		
80	0.019 ± 0.002	6.80 ± 0.47		
40			0.065 ± 0.003	9.87 ± 0.86
−40			0.050 ± 0.003	6.95 ± 0.70
−80	0.020 ± 0.002	10.78 ± 0.79		
−100	0.021 ± 0.0006	12.74 ± 0.60		
−120	0.016 ± 0.0004	7.20 ± 1.07		

**Table 4:** Capacitance variation in POPC:Ch PLM. Mean values of the membrane capacitance (C ± SE) calculated at T0, T1, T2, and T3, for which meanings are reported in the legend to Table 3. The mean value was obtained from at least four experiments.

Time	C ± SE µF/cm^2^ [Res] = 10 µM	C ± SE µF/cm^2^ [Res] = 20 µM
T0	0.30 ± 0.02	0.30 ± 0.02
T1	0.11 ± 0.002	0.12 ± 0.002
T2	0.29 ± 0.01	0.28 ± 0.01
T3	0.30 ± 0.02	0.31 ± 0.01

**Table 5:** Capacitance variation in DOPS:DOPE PLM. Mean values of the membrane capacitance (C ± SE) calculated at T0, T1, T2, and T3, for which meanings are reported in the legend to Table 3. The mean value was obtained from at least six experiments.

Time	C ± SE µF/cm^2^ [Res] = 10 µM	C ± SE µF/cm^2^ [Res] = 20 µM
T0	0.26 ± 0.01	0.28 ± 0.01
T1	0.10 ± 0.003	0.11 ± 0.002
T2	0.10 ± 0.002	0.11 ± 0.002
T3	0.10 ± 0.003	0.12 ± 0.001
